# Estimation of potato canopy leaf water content in various growth stages using UAV hyperspectral remote sensing and machine learning

**DOI:** 10.3389/fpls.2024.1458589

**Published:** 2024-11-14

**Authors:** Faxu Guo, Quan Feng, Sen Yang, Wanxia Yang

**Affiliations:** College of Mechanical and Electrical Engineering, Gansu Agriculture University, Lanzhou, China

**Keywords:** hyperspectral remote sensing, inversion mapping, machine learning, leaf water content, potato, spectral feature extraction unmanned aerial vehicle

## Abstract

To ensure national food security amidst severe water shortages, agricultural irrigation must be reduced through scientific innovation and technological progress. Efficient monitoring is essential for achieving water-saving irrigation and ensuring the sustainable development of agriculture. UAV hyperspectral remote sensing has demonstrated significant potential in monitoring large-scale crop leaf water content (LWC). In this study, hyperspectral and LWC data were collected for potatoes (*Solanum tuberosum*) during the tuber formation, growth, and starch accumulation stage in both 2021 and 2022. The hyperspectral data underwent mathematical transformation by multivariate scatter correction (MSC) and standard normal transformation (SNV). Next, feature spectral bands of LWC were selected using Competitive Adaptive Reweighted Sampling (CARS) and Random Frog (RF). For comparison, both the full-band and feature band were utilized to establish the estimation models of LWC. Modeling methods included partial least squares regression (PLSR), support vector regression (SVR), and BP neural network regression (BP). Results demonstrate that MSC and SNV significantly enhance the correlation between spectral data and LWC. The efficacy of estimation models varied across different growth stages, with optimal models identified as MSC-CARS-SVR (R^2^ = 0.81, RMSE = 0.51) for tuber formation, SNV-CARS-PLSR (R^2^ = 0.85, RMSE = 0.42) for tuber growth, and MSC-RF-PLSR (R^2^ = 0.81, RMSE = 0.55) for starch accumulation. The RPD values of the three optimal models all exceed 2, indicating their excellent predictive performance. Utilizing these optimal models, a spatial distribution map of LWC across the entire potato canopy was generated, offering valuable insights for precise potato irrigation.

## 1 Introduction

As global agriculture faces increasing pressure from water scarcity, efficient water management has become more critical than ever. The Food and Agriculture Organization (FAO) of the United Nations projects that by 2050, the world will need to produce 60% more food to meet the demands of a population expected to reach 9.7 billion, all while grappling with increasingly limited water resources (Lakhiar et al., 2024). In this context, precision irrigation emerges as a key strategy to optimize water usage, minimize waste, and ensure sustainable agricultural productivity. Water plays an indispensable role in the growth and development of crops (Féret et al., 2019; Liu et al., 2022; Li et al., 2022a). Crops rely on water for photosynthesis, transpiration, and the synthesis and decomposition of organic matter (Sun et al., 2015; Meiyan et al., 2022). Obtaining crop water information quickly and accurately is vital for timely crop irrigation and yield improvement, especially in regions prone to water scarcity. Efficient water use is critical in agriculture, especially in water-scarce regions. In modern precision agriculture, technologies like UAV hyperspectral remote sensing provide a promising solution for monitoring crop water content in real-time. Among all crop tissues, leaves exhibit the most vigorous metabolism and serve as the primary site for photosynthesis (Zhou et al., 2020; Zhang et al., 2021). Analyzing leaf water content (LWC) is critical for assessing crop moisture status, as LWC level indicate the degree of crop water deficiency. Traditional methods of measuring crop moisture, such as drying and distillation, are accurate but time consuming and energy intensive (Roberto et al., 2018; Wang et al., 2021). Therefore, more efficient monitoring methods are needed for precise agricultural management. When crops suffer from severe water shortages, timely irrigation becomes a challenge.

In the past few decades, satellite-based hyperspectral remote sensing technology has been extensively studied in large-scale regional agricultural monitoring such as crop nitrogen content (Zheng et al., 2022), crop chlorophyll content (Xie and Yang, 2020), and crop biomass (Jia et al., 2019). In China, the cultivated land area of a farmer is usually no more than a few acres. On this scale, the granularity of satellite remote sensing is too rough, and it is difficult for a farmer to obtain remote sensing data in time. As a result, this method cannot provide farmers with timely information on drought conditions and irrigation guidance. In recent years, with the development of technology, the price of unmanned aerial vehicle (UAV) has been becoming cheaper. Chinese farmers have used UAV for many field operations, such as spraying and short-distance transport. Compared with satellites, the remote sensing mode of UAV + hyperspectral sensing has many advantages such as fine granularity, convenience, and flexibility. The combination of the two technologies can make precision agriculture more practical in China and can provide farmers with a scientific basis for precision irrigation based on LWC measurements (Zhang et al., 2021; He et al., 2023). UAV hyperspectral remote sensing offers farmers timely, high-resolution data that can improve precision irrigation strategies. For example, recent studies demonstrated its effectiveness in optimizing water management in crops like maize and wheat, leading to measurable water savings and increased yields (Mohite et al., 2022; Luo et al., 2024).

The widespread adoption of hyperspectral remote sensing technology has enabled its extensive use in monitoring plant water content (Suárez et al., 2009; Raj et al., 2021), and most studies have focused on the infrared region at wavelengths greater than 900 nm. Maes and Steppe (2019) discovered that the vibrational movements of water and other molecules containing O-H groups in plants result in spectral absorption peaks occurring near 970 nm, 1200 nm, 1450 nm, 1940 nm, and 2500 nm in the spectral reflectance of plants. Zhang et al. (2014) investigated the optimal spectral indicators for determining LWC. Their findings demonstrated that the regression model for leaf water content based on the normalized difference spectral index NDSI (R1222, R2264) and the ratio spectral index RSI (R2264, R1321), is closely aligned with the measured and estimated values. Sun et al. (2021) utilized the Fractional Order Savitzky-Golay Derivative (FOSGD) to preprocess the hyperspectral reflectance data of maize leaves spanning from 900 nm to 1700 nm. They employed Variable Importance in Projection (VIP), Competitive Adaptive Reweighted Sampling (CARS), and Random Frog (RF) methods to identify sensitive wavelengths. They established a maize leaf water content estimation model based on Partial Least Squares. The results indicated that the FOSGD-CARS-PLS or FOSGD-RF-PLS model can effectively predict the LWC of maize. Although near-infrared spectra above 900 nm exhibit high reliability in measuring vegetation moisture, the instruments acquiring these spectra are characterized by their high cost, making it unaffordable for the ordinary farmers.

Visible and near-infrared spectroscopy (VIS-NIR) has been extensively employed for estimating leaf water content (LWC) due to its cost-effectiveness and wide accessibility. Previous studies have demonstrated that vegetation indices derived from VIS-NIR reflectance can effectively detect changes in LWC across a variety of crops (Suárez et al., 2008; Kovar et al., 2019). For instance, Ishikawa et al. (2013) found that spectral bands in the 650–690 nm range were strongly correlated with LWC in diverse leaf samples, underscoring the reliability of this method for water content estimation. More recent research by Wang et al. (2023) utilized partial least squares (PLS) models to predict moisture content in fresh tea leaves, integrating spectral data to provide practical applications in real-time monitoring systems. Similarly, Duarte-Carvajalino et al. (2021) applied hyperspectral imaging techniques to predict potato leaf water content, achieving high accuracy under controlled experimental conditions. Nevertheless, these studies predominantly focus on controlled environments, where factors such as temperature, humidity, and lighting are strictly regulated. As a consequence, the models developed may exhibit reduced performance in field conditions, where environmental variability introduces significant challenges.

Additionally, many studies have constructed spectral models specific to particular growth stages, thereby limiting their applicability across the entire crop life cycle (Panigrahi and Das, 2018). Given that crop spectral characteristics evolve with growth stage, health status, and environmental conditions, models developed for a single growth stage may fail to capture the full extent of LWC variation throughout the entire growth period. While some research, such as that by Liu et al. (2015), has compared models across multiple growth stages, these models often require further validation to ensure their robustness under diverse conditions. Consequently, there remains a critical gap in the development of LWC estimation models that can be reliably applied across multiple growth stages, particularly for crops like potatoes, which exhibit varying water demands at different phenological phases (Sudu et al., 2022).

Potato, along with rice, wheat, and corn, is one of the major food crops globally (Zhang et al., 2017). The vitality and water content of leaf play a crucial role in ensuring a nation’s food security (Wijesinha-Bettoni and Mouillé, 2019). Especially in water-scarce regions such as Gansu Province, potatoes, as a key crop, have significant water requirements, making them an ideal subject for research on improving irrigation efficiency. Although numerous studies have applied hyperspectral remote sensing to monitor crop water content, relatively few have specifically focused on potatoes, particularly under real field conditions. Additionally, most existing models are limited to a single growth stage, neglecting the variations in moisture content across different stages of growth. As shown in **Table 1**, these studies face challenges such as limited spatial coverage, insufficient data acquisition speed, and a lack of validation in real field conditions. This table summarizes the key differences between our research and previous studies, highlighting how our work addresses these limitations. This study aims to address the limitations of previous research by developing and validating a potato LWC estimation model across multiple growth stages using UAV-based hyperspectral remote sensing combined with machine learning algorithms. By focusing on critical growth stages such as tuber formation, growth, and starch accumulation, this research offers valuable insights for optimizing irrigation strategies and advancing precision agriculture techniques in water-scarce regions.

**Table 1:** Overview of hyperspectral remote sensing methods and their limitations in existing studies.

Reference	Cereals	Remote Sensing Platforms	Spectral Range (nm)	Regressors	Limitations	Improvements in This Study	
Liu et al. (2022)	Winter Wheat	Ground-Based Platform	350–2500	MLR, SVR, PLSR	Limited spatial coverage	Utilized high-resolution UAV for extensive coverage
Li et al. (2022a)	Winter Wheat	Ground-Based Platform	350–1350	GPR, CART, ANN	Limited spatial coverage	Utilized high-resolution UAV for extensive coverage
Feng et al. (2022)	Winter Wheat	UAV-Based Platform	450–950	MLR, PLSR, RF	Focused only on a single growth stage	Monitored multiple growth stages for potatoes
Li et al. (2022b)	Winter Wheat	UAV-Based Platform	400–1000	SVR, LRR, RF, GPR	Focused only on a single growth stage	Monitored multiple growth stages for potatoes
Sun et al. (2015)	Corn	Ground-Based Platform	900–1700	PLSR, MLR, BP	Not validated in real field conditions	Collected data in actual field conditions
Sun et al. (2021)	Corn	Ground-Based Platform	900–1700	PLSR, MLR	Data collection conditions determined	Collected data in actual field conditions
Ndlovu et al. (2021)	Maize	UAV-Based Platform	475, 560, 668, 717, 840	RF, SVR, PLSR	Focused only on a single growth stage	Monitored multiple growth stages for potatoes
Sudu et al. (2022)	Summer Maize	UAV-Based Platform	450–998	PLSR, RF, XGBoost	Multi-Growth stage mixed modeling	Independent modeling for each growth stage
Tunca et al. (2023)	Sorghum	Ground-Based Platform	325–1075	RF, SVR, XGBoost	Limited spatial coverage	Utilized high-resolution UAV for extensive coverage
Krishna et al. (2019)	Rice	Ground-Based Platform	350–2500	PLSR, MLR, ANN; RF, SVR	Limited spatial coverage	Utilized high-resolution UAV for extensive coverage
Elsherbiny et al. (2021)	Rice	Ground-Based Platform	874–1734	BP, RF, PLSR	Data collection conditions determined	Collected data in actual field conditions
Chen et al. (2020a)	Cotton	UAV-Based Platform	490, 550, 680, 720, 800, 900	MLR	Focused only on a single growth stage	Monitored multiple growth stages for potatoes
Duarte-Carvajalino et al. (2021)	Potato	Ground-Based Platform	400–1000	RF, XGBoost, MLP, CNN, SVR	Limited spatial coverage	Utilized high-resolution UAV for extensive coverage
Suyala et al. (2024)	Potato	Ground-Based Platform	337–2521	PLSR, SVR, BP	Limited spatial coverage	Utilized high-resolution UAV for extensive coverage
Guo et al. (2023)	Potato	UAV-Based Platform	400–1000	PLSR, MLR	Multi-Growth stage mixed modeling	Independent modeling for each growth stage

MLR denotes Multiple Linear Regression, GPR denotes Gaussian Process Regression, CART denotes Classification and Regression Tree, ANN denotes Artificial Neural Network Regression, RF denotes Random Forest Regression, LRR denotes Linear Ridge Regression, and XGBoost denotes Extreme Gradient Boosting Regression.

## 2 Materials and methods

### 2.1 Overview of the study area

**Figure 1:**
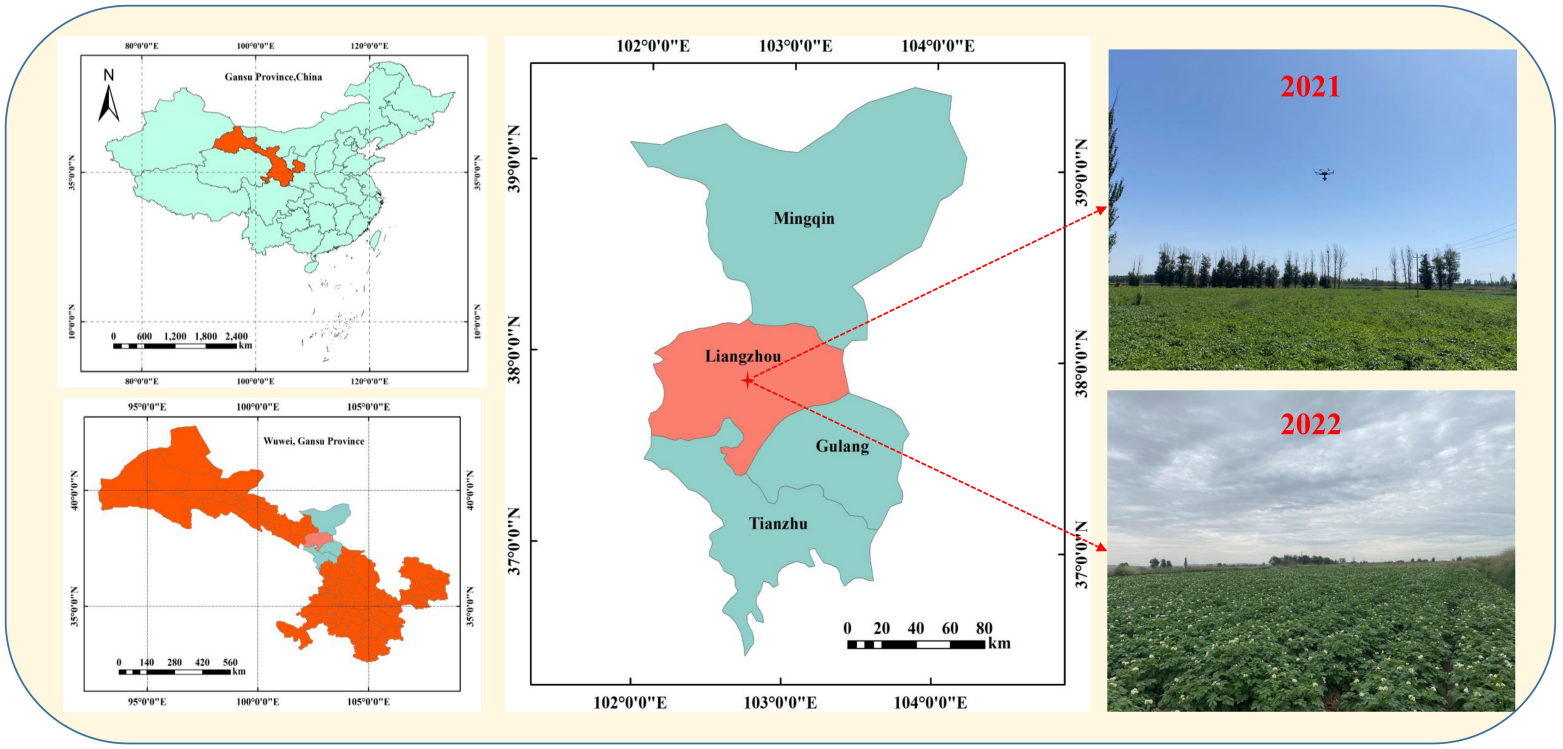
Description of the geographic location of the study area.

Potato field trials were conducted in Huangyang Town, Liangzhou District, within Wuwei City, from May to October in both 2021 and 2022. Huangyang Town, located in the Hexi Irrigation District of Gansu Province (37°81^′^49^′′^ *N*, 102°92^′^38^′′^ *E*) (**Figure 1**), is a significant area for potato cultivation. The area is situated at an altitude of 1660 m and lies in the eastern part of Hexi Corridor. It has a continental temperate arid climate with a 150-day frost-free period, averaging an annual temperature of 7.2°C and receiving 160 mm of annual precipitation. The soil in this area is grey calcareous with sandy loam texture and is high saline, PH value of 7.82. The main crops cultivated are corn, wheat, and potatoes. The potato variety ‘Qingshu9’ was planted in both years, courtesy of Gansu Academy of Agricultural Sciences. ‘Qingshu 9’ is a medium-to late-maturing fresh potato variety, with an average growth period of approximately 115 days from emergence to maturity. This variety is characterized by its high yield, resistance to late blight and scab, and adaptability to local climatic conditions.

Prior to planting, two types of fertilizers were applied: diammonium phosphate (46%P_2_O_5_, 18%N) at a rate of 400 kg/hm^2^, to provide the necessary phosphorus and nitrogen for the early growth stages, and a Western compound fertilizer (15%N, 15%P_2_O_5_, 15%K2O) at a rate of 750 kg*/*hm^2^, to ensure adequate nutrient supply throughout the reproductive period. Both fertilizers were incorporated into the soil in a single application before sowing. Irrigation was carried out using an under-membrane drip system, with drip pipes buried 6cm deep (Aziz et al., 2021). The irrigation system used drip pipes with a diameter of 16 mm (*φ*16) and emitters for water delivery, while the branch pipe was a 50 mm diameter (*φ*50) PE pipe with a pressure resistance of over 0.5MPa. To minimize water evaporation and maintain soil moisture, the soil surface was covered with a black polyethylene film, 90cm wide and 0.012mm thick. Throughout the growth cycle of the potato crop, the total irrigation volume ranged from 2100 to 2300m^3^/hm^2^. In 2021, irrigation was performed 10 times, while in 2022, it was conducted 11 times, with irrigation intervals of 10−12 days. The irrigation frequency was adjusted according to3the potato2 growth stage and soil moisture levels, with each irrigation delivering between 190 and 250 m/hm. Field management tasks such as sowing, fertilizing, weeding, spraying pesticides, and other farming practices were carried out according to local agricultural practices.

### 2.2 Hyperspectral image acquisition and processing

**Figure 2:**
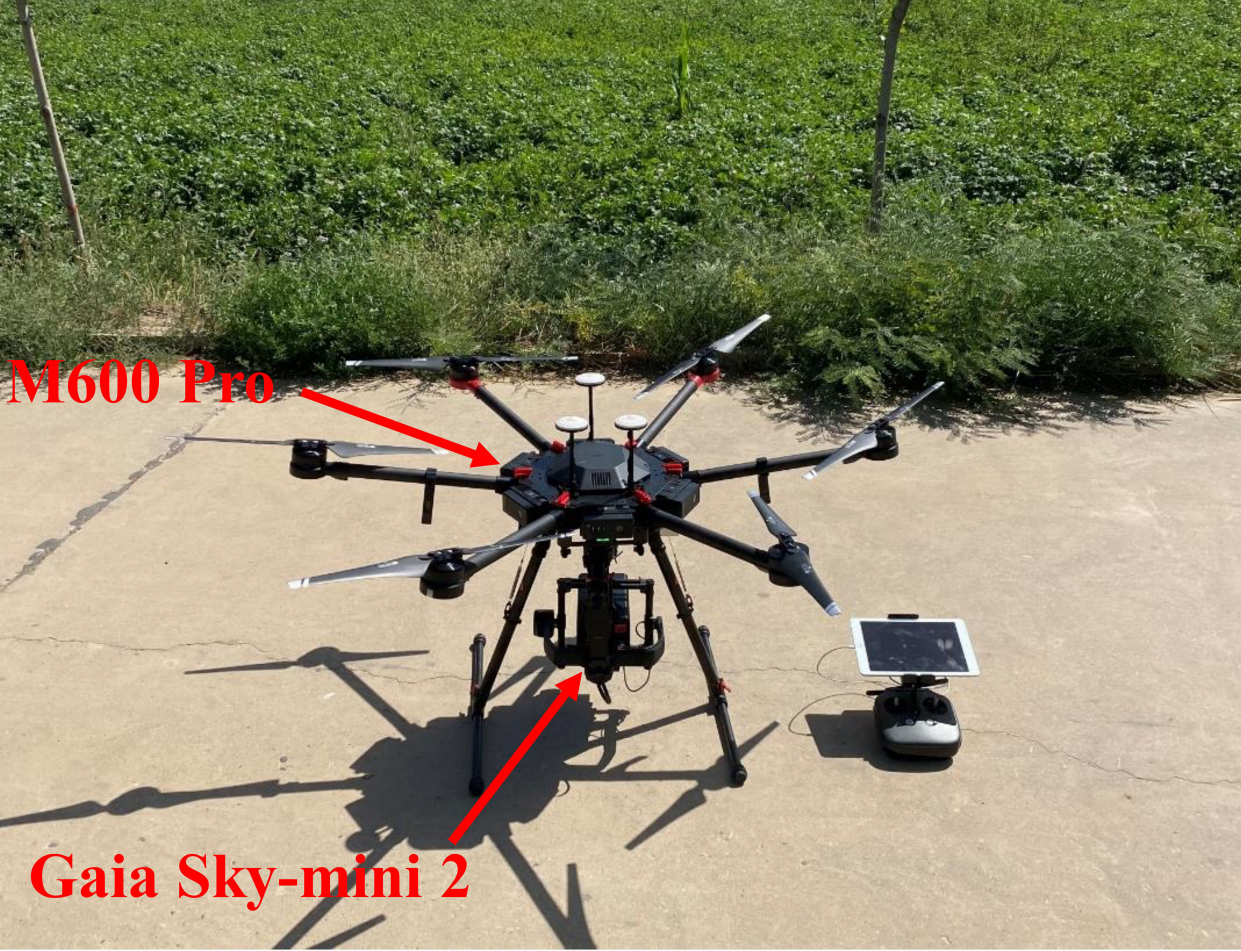
UAV imaging hyperspectral system.

Potatoes were planted on May 6, 2021, and May 10, 2022, and harvested on October 3, 2021, and October 8, 2022, respectively. The tuber formation stage began 65 days after planting and lasted for approximately 30 days. The tuber growth phase commenced 95 days after planting and continued for about 25 days, while the starch accumulation phase started 120 days after planting and lasted for around 30 days (Aziz et al., 2021). The study conducted six field trials at three crucial growth stages of potato: July 28, 2021 (S1 - tuber formation stage), August 19 (S2 - tuber growth stage), August 30 (S3 - starch accumulation stage), and August 1, 2022 (S1 - tuber formation stage), August 17 (S2 - tuber growth stage), August 31 (S3 - starch accumulation stage). Hyperspectral images were obtained using the DJI M600 Pro^®^ hexacopter drone, equipped with the Gaia Sky-mini 2^®^ imaging spectrometer (Jiangsu Dualix Spectral Imaging Technology Co., Ltd, China) (**Figure 2**). The Gaia Sky-mini 2 features a built-in push-scan imaging system with a spectral range of 400 to 1000 nm and a spectral resolution of 3.5 nm. It has a full-frame pixel resolution of 1392×1040 and weighs approximately 1 kg. The device employs a surface-array detector oriented perpendicularly to the direction of movement, allowing it to perform a two-dimensional spatial scan as the motion platform advances. The DJI M600 Pro hexacopter, when unloaded, has a flight time of approximately 35 minutes per battery. Under a maximum load of 6 kg, its battery life is reduced to around 16 minutes. To ensure high-quality hyperspectral images, all six experiments were conducted between 11:00 a.m. and 1:00 p.m. local time, following a fixed flight path on days with stable sunlight intensity and clear, cloudless weather. The drone operated at a flight height of 100 m, with a 22° scanning field of view, a high-altitude resolution of 0.039m, 80% lateral overlap, and 60% longitudinal overlap between image data. Before the UAV’s departure, the hyperspectral imager underwent radiometric calibration using a whiteboard. The pre-processing of the hyperspectral data primarily involves image correction, stitching, and reflectance extraction (Guo et al., 2023).

### 2.3 Measurement and statistics of LWC data

Potato leaf sampling and hyperspectral imaging were synchronized. Sampling points were uniformly distributed based on the study and potato planting areas. The center coordinates of each observation were located and recorded using a handheld high-precision GPS sampling device with a positioning accuracy of< 5 cm. Twenty fully expanded and undamaged leaves were collected from each sampling point in the canopy. The collected leaves were sealed, and their fresh weight (*W*_1_, g) was measured in the laboratory using a precision electronic balance (JA3003, Shanghai Hengping Instrument and Meter Co., Ltd., China). Next, the leaves from each sampling point were dried in a laboratory oven at 105°C for 30 min and then further dried at 80°C until a constant weight was achieved (Xu et al., 2022). The dry weight of the leaves (*W*_2_, g) was recorded. The LWC (%) of the potato was calculated by Equation 1:


(1) $$LWC=\frac{W_{1}-W_{2}}{W_{1}}\times 100\%$$

This study measured the potato LWC data at three critical growth stages. At stage S1, 50 samples were collected each year, while at stages S2 and S3, 55 samples were collected each year. A total of 320 samples were collected during the experimental phase.

### 2.4 Hyperspectral data transformation

During hyperspectral data processing, specific spectral transformations can be used to mitigate the effects of environmental factors and interferences, improve the signal-to-noise ratio, and make spectral forms more sensitive to potato LWC (Yang et al., 2023). In this study, several mathematical transformations were applied to the raw spectra, resulting in six spectral data types: raw reflectance (R), multiple scattering correction (MSC), standard normal variate (SNV), reciprocal transformation (RT), logarithm of the reciprocal [(Lg(1/R)], and first derivative (FD).MSC is a normalization technique that reduces baseline drift, improves the signal-to-noise ratio, and better reveals differences and similarities between samples. It is commonly used to eliminate the effects of scatter on spectral data (Chen et al., 2019; Sun et al., 2020). SNV primarily eliminates the effects of solid particle size, surface scattering, and optical path variations on spectra. Elimination of baseline drift and enhancement spectral signal characteristics contribute to improved accuracy and reliability in spectral analysis (Genkawa et al., 2015; Tao et al., 2020). RT and Lg(1/R) help to enhance features in low reflectance regions, making subtle changes more detectable. Meanwhile, FD highlights minute spectral changes, such as the positions of reflectance peaks and valleys, although it may amplify noise in regions with weaker signals (Sonobe and Hirono, 2022).

### 2.5 Extraction of hyperspectral feature band

Considering the large number of collected hyperspectral bands, numerous redundant and interfering bands exist. Extracting the feature band related to potato LWC based on the potato hyperspectral reflectance data is crucial. This study utilized two hyperspectral feature band extraction algorithms: CARS and RF.

The CARS algorithm (Sun et al., 2021; Xing et al., 2021) is based on in the evolutionary principle of “survival of the fittest”. It integrates partial least squares (PLS) with CARS technology by using the absolute value percentage of the PLS modeling coefficients as a measure of significance for the target variables. And then it selects wavelengths with substantial coefficients, and discarding those with low weights. The process uses Monte Carlo Sampling (MCS), exponential decay functions, and Adaptive Weighted Sampling (ARS) to acquire an initial subset of wavelengths for further screening. Multiple iterations of the CARS algorithm result in a set of wavelengths closely linked to the target attributes. This produces a high-performance feature wavelength set for regression modeling. The comprehensive approach improves the accuracy and reliability of spectral analysis, effectively selecting the best modeling wavelength combinations. The RF algorithm (Li et al., 2012; Hu et al., 2015) is a heuristic feature selection algorithm that is highly suitable for spectral data. The RF algorithm mimics the random yet systematic search behavior of a frog ‘leaping’ between different subsets of features. Each ‘leap’ represents the algorithm’s movement from one potential feature subset to another, selecting subsets based on performance. By continuously performing crossbreeding, mutation, and selection operations, the algorithm gradually optimizes the feature subset to excel in the given task. This method allows the algorithm to explore various feature combinations in the search space to identify the best feature set, thereby improving the model’s performance.

### 2.6 Machine learning modeling

Following the aforementioned processes on the hyperspectral data, three regression algorithms (PLSR, SVR and BP) of machine learning were used to construct a potato LWC estimation models. PLSR (Cheng and Sun, 2017) is a traditional linear regression technique that conducts principal component analysis on the explanatory and response variables to identify a new feature space that optimizes their covariance. This method effectively handles multicollinearity, is suitable for high-dimensional data, and can handle multiple response variables. PLSR ensures that each component is associated with the target, allowing for multi-level regression analysis by retaining different numbers of components to achieve more robust predictions and a deeper understanding of the relationship between independent and dependent variables (Burnett et al., 2021). The model training process involved optimizing the number of principal components, which in this study was varied between two and two-thirds of the total number of features.

SVR (Virnodkar et al., 2020) is based on kernel statistical theory and transforms the sample space into a high-dimensional or infinite-dimensional feature space through nonlinear mapping. This conversion turns the initially nonlinear separable problem in the sample space into a linear separable problem in the feature space. SVR can improve robustness against noise and outliers, exhibits nonlinear modeling capabilities, excels in high-dimensional space, and provides strong generalization performance, making it suitable for various regression problems. In this study, a radial basis function was employed, with the two parameters to be optimized being the penalty coefficient (C) and the kernel function parameter (γ). A grid search approach was utilized to determine the optimal values for these parameters, where the search range for C was from 0.5 to 500, and for γ, from 0.0001 to 0.05.

The Backpropagation (BP) neural network (Li et al., 2016) is widely used for nonlinear modeling and data prediction. It consists of input, output, and intermediate hidden layers. The learning process involves two key steps: forward propagation and backpropagation. During forward propagation, the input data is processed layer by layer, starting with the input layer, then the hidden layer, and finally the output layer. In case of an error between the predicted result of the output layer and the actual data, the backpropagation process is initiated. By using the gradient descent method, the backpropagation algorithm methodically adjusts the weights of each neuron layer by layer, continuing until the error aligns with the predetermined criteria. In the present research, it was necessary to determine the number of hidden layers and the corresponding number of neurons in each layer. Two hidden layers with the same number of neurons were employed. The empirical formula (Equation 2) was applied to define an appropriate range for the number of nodes in the hidden layers. The optimal number of nodes in each hidden layer was then selected using the grid-search method, with 1,000 iterations, a learning rate of 0.01, and a training objective of 1×10^−6^.


(2) $$q=\sqrt{k+m}+\alpha$$

where *q* denotes the number of nodes in the hidden layer, *k* represents the number of input layer units, *m* indicates the number of output layer units, and *α* is a constant in the range [1, 10].

### 2.7 Model evaluation methods

The regression algorithms employed in this research were implemented in the Python 3.6.13 environment using the scikit-learn 0.23.2 or TensorFlow 2.1.0 frameworks. Due to the limited number of samples available for a single growth stage, the constructed models were validated through leave-one-out-cross validation (Wong, 2015). During the cross-validation process, each sample was iteratively used as a test set. Model parameters were determined by a comprehensive comparison of multiple training iterations, providing results that are considered the closest approximation to the expected value derived from training on the entire dataset. We use the coefficient of determination (R^2^), root mean square error (RMSE) and relative analytical error (RPD) as evaluation metrics to assess model performance. R^2^ (closer to 1) measures how well the model fits the data, while RMSE (closer to 0) quantifies the spread of predicted values around the regression line. A higher R^2^ and a lower RMSE indicate greater precision in model estimation. The RPD is used to assess the predictive ability of a model. It is defined as the ratio of the standard deviation of the sample to the RMSE. When RPD< 1.4, the model is considered unable to predict the samples accurately. If 1.4 ≤ RPD< 2, the model is regarded as moderately effective and can be used for rough assessments. For RPD ≥ 2, the model is considered to have excellent predictive capability (Zhu et al., 2020). The calculation of R^2^, RMSE, and RPD are shown in Equations 3–5, respectively.


(3) $$R^{2}=1-\frac{\sum_{i=1}^{n}{\left(y_{m}-y_{p}\right)}^{2}}{\sum_{i=1}^{n}{\left(y_{m}-\bar{y}\right)}^{2}}$$

(4) $$RMSE=\sqrt{\frac{1}{n}\sum_{i=1}^{n}{\left(y_{m}-y_{p}\right)}^{2}}$$

(5) $$RPD=\frac{S_{y}}{RMSE}=\frac{\sqrt{\frac{1}{n-1}\sum_{i=1}^{n}{\left(y_{m,i}-{\bar{y}}_{m}\right)}^{2}}}{RMSE}$$

Where *n* is the sample size; $y_{m}$ and $y_{p}$ are the actual and predicted values of potato LWC, respectively; $\bar{y}$ is the mean value of the actual potato LWC; $S_{y}$ is the standard deviation of the measured value of potato LWC.

## 3 Results and analysis

### 3.1 Mathematical statistics of collection sample

**Figure 3:**
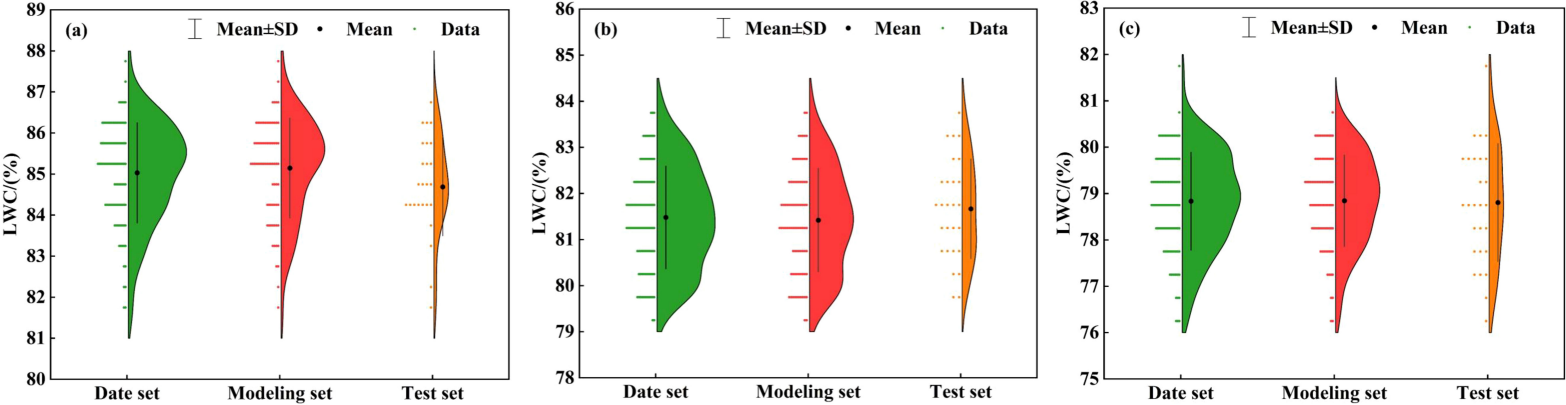
Distribution of potato LWC at different growth stages, **(A)** tuber formation stage S1, **(B)** tuber growth stage S2, **(C)** starch accumulation stage S3.

To ensure that the modeling and test data sets better capture the entire dataset’s distribution, to mitigate bias from specific data distributions, and to improve the generalizability of the model, we used a random splitting method, allocating 75% of the samples for modeling and 25% for testing. For the potato growth stage S1, 75 samples were selected for the modeling data set and 25 for the test data set. Likewise, for the S2 and S3 growth stages, 83 samples were selected for the modeling data set, and 27 in the test data set. **Figure 3** shows the sample distribution in the modeling and test data sets across the three growth stages of potato. As shown in **Figure 3**, the mean and standard deviation (SD) values of the sample points of the three different growth stages can be obtained. In the S1 stage, the distribution of LWC in the modeling data set ranges from 81.53% to 87.91% (SD = 1.22), while in the test data set, it ranges from 81.70% to 86.62% (SD = 1.19). In the S2 stage, the distribution of LWC in the modeling data set ranges from 79.28% to 83.99% (SD = 1.12), while in the test data set, it ranges from 79.90% to 83.84% (SD = 1.08). In addition, at the S3 stage, the distribution of LWC in the modeling data set ranges from 76.28% to 80.72% (SD = 0.99), while in the test data set, it ranges from 76.40% to 81.58% (SD = 1.27). The mean and standard deviation (SD) values between the modeling and test datasets for the three growth stages show minor variances, indicating a reasonable division of the data set.

### 3.2 Potato hyperspectral features

#### 3.2.1 Hyperspectral transformation and feature analysis

**Figure 4:**
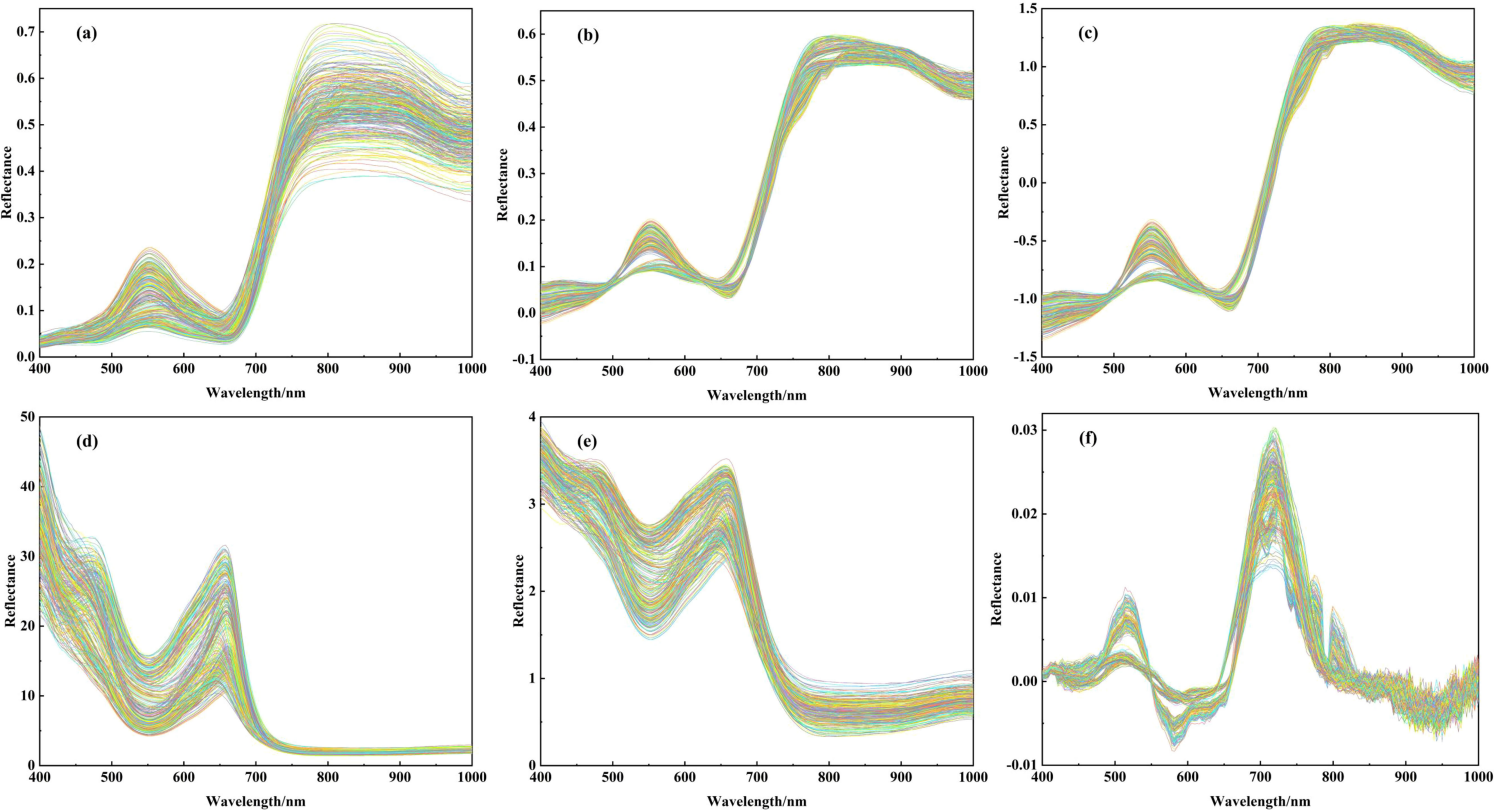
Raw and mathematically transformed spectra of potato, **(A)** raw spectra, **(B)** multiple scattering correction transformation spectrum, **(C)** standardized normal variate transformation spectrum, **(D)** inverse transformation spectrum, **(E)** ogarithm of the reciproca transformation spectrum, **(F)** first derivative transformation spectrum.

**Figure 4** shows the original spectral curves and their mathematical transformations for the potato samples. As can be seen from **Figure 4A**, there are obvious differences in the raw reflectance (R) between the samples, and there are phenomena such as baseline shift and tilt between the spectra. These observations can be attributed to light scattering from the potato canopy and changes in the optical path length. After processing with by MSC (**Figure 4B**) and SNV (**Figure 4C**), the discrepancies in reflectance are significantly decreased. The spectra show higher concentration and consistent spectral curve characteristics. These results provide the evidence that the two spectral transformation methods can effectively address spectral shifts, eliminate background interference and noise, and enhance spectral features. As a result, feature wavelengths can be identified with greater accuracy and precision. The RT transform (**Figure 4D**) effectively enhances the spectral features in regions of low reflectance, highlighting subtle changes that are less apparent in the original spectrum. In contrast, the Lg(1/R) transform (**Figure 4E**) emphasizes spectral variations while preserving the overall trend of the spectrum, making it suitable for analyzing subtle differences. The FD transform (**Figure 4F**) effectively highlights small changes in the spectrum, such as the positions of reflectance peaks and valleys, thereby reducing background interference and improving the accuracy of feature extraction. However, a burr phenomenon is observed beyond 800 nm in **Figure 4F**, primarily due to weak signal strength in this spectral region, which results in noise amplification during the derivative transformation process.

#### 3.2.2 Analysis of the correlation between potato canopy reflectance and LWC

**Figure 5:**
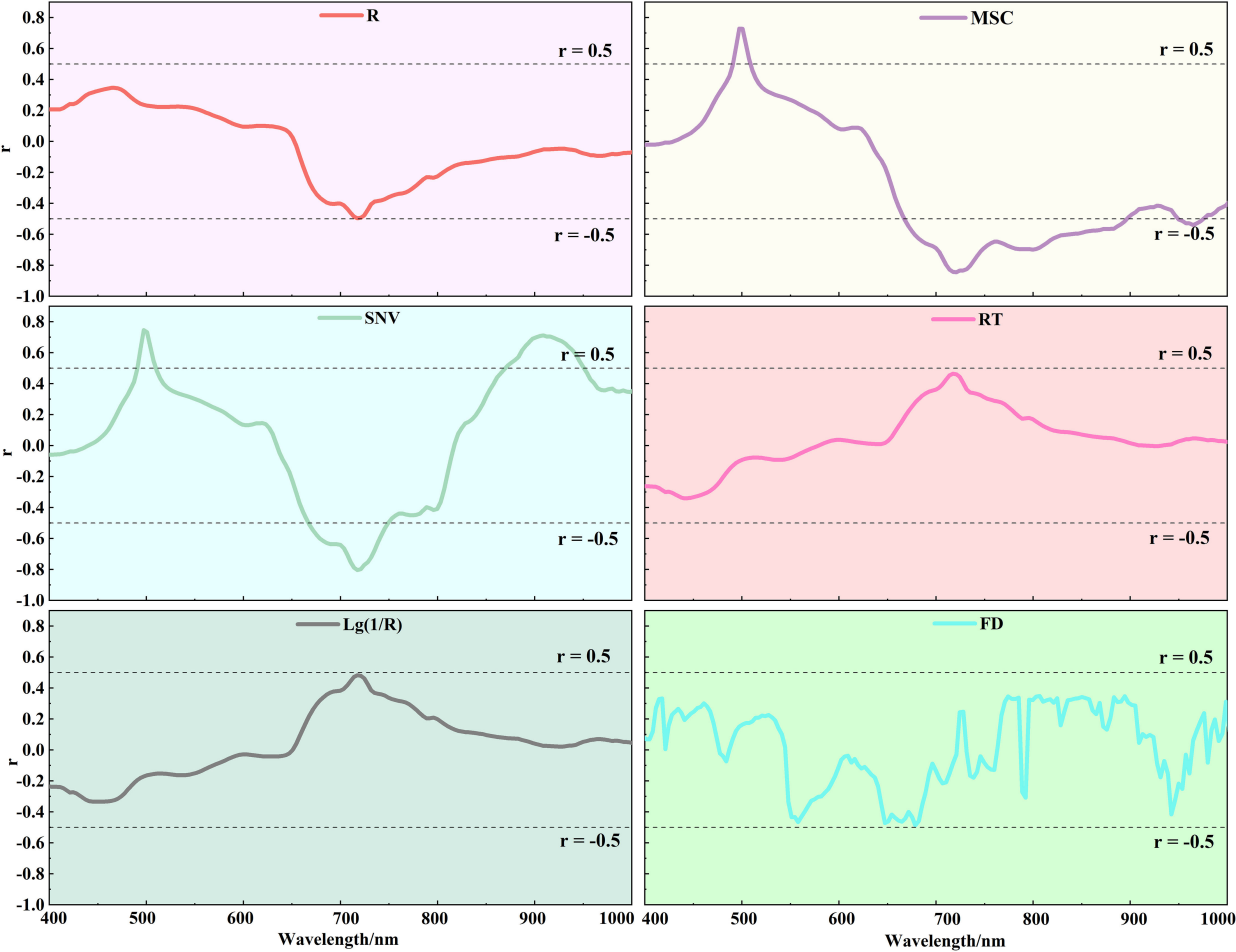
Correlation analysis of potato LWC with different transformed spectra.

Based on the actual measured data of LWC in three critical growth stages of potato, the correlation between the reflectance of each band before and after hyperspectral mathematical transformation with LWC is analyzed during the entire growth period of potato, and the results are shown in **Figure 5**. It can be observed that the correlation coefficients *r* for MSC and SNV show an increase compared to R. *r* between R and LWC ranges from -0.50 to 0.35, while for MSC and LWC, it ranges from -0.84 to 0.73, and for SNV and LWC, it ranges from -0.80 to 0.75. The trend of change in *r* between the reflectance of each band and LWC before and after spectral transformation remains consistent in the range of 500-725 nm. The absolute value of the negative correlation coefficient r reaches its highest point for each transformation at approximately 725 nm, in fact, in this band, all three transformations show the greatest negative correlation with LWC. This suggests that the correlation between spectral features and LWC is better revealed after applying MSC and SNV transformations in this spectral region. In contrast, the correlation curves of RT, Lg(1/R), and FD are smoother, with relatively small fluctuations in the correlation coefficients. Although these transformations provide unique feature information in specific bands (e.g., low reflectance regions), they do not show a significant enhancement in correlation within critical bands (e.g., near 725 nm). This is particularly evident for the FD transform, which shows noise amplification in bands above 800 nm, leading to large fluctuations at high frequencies. As shown in **Figure 5**, MSC and SNV spectral data are more effective in revealing the relationship between LWC and spectral reflectance. While the RT, Lg(1/R), and FD transforms offer some information gain, their correlation performance is limited in key bands and is more susceptible to noise interference in certain regions. Therefore, R, MSC, and SNV spectral data are selected for subsequent experiments to improve the accuracy of the analysis and the reliability of the model.

### 3.3 Selection of feature band for potato LWC

**Figure 6:**
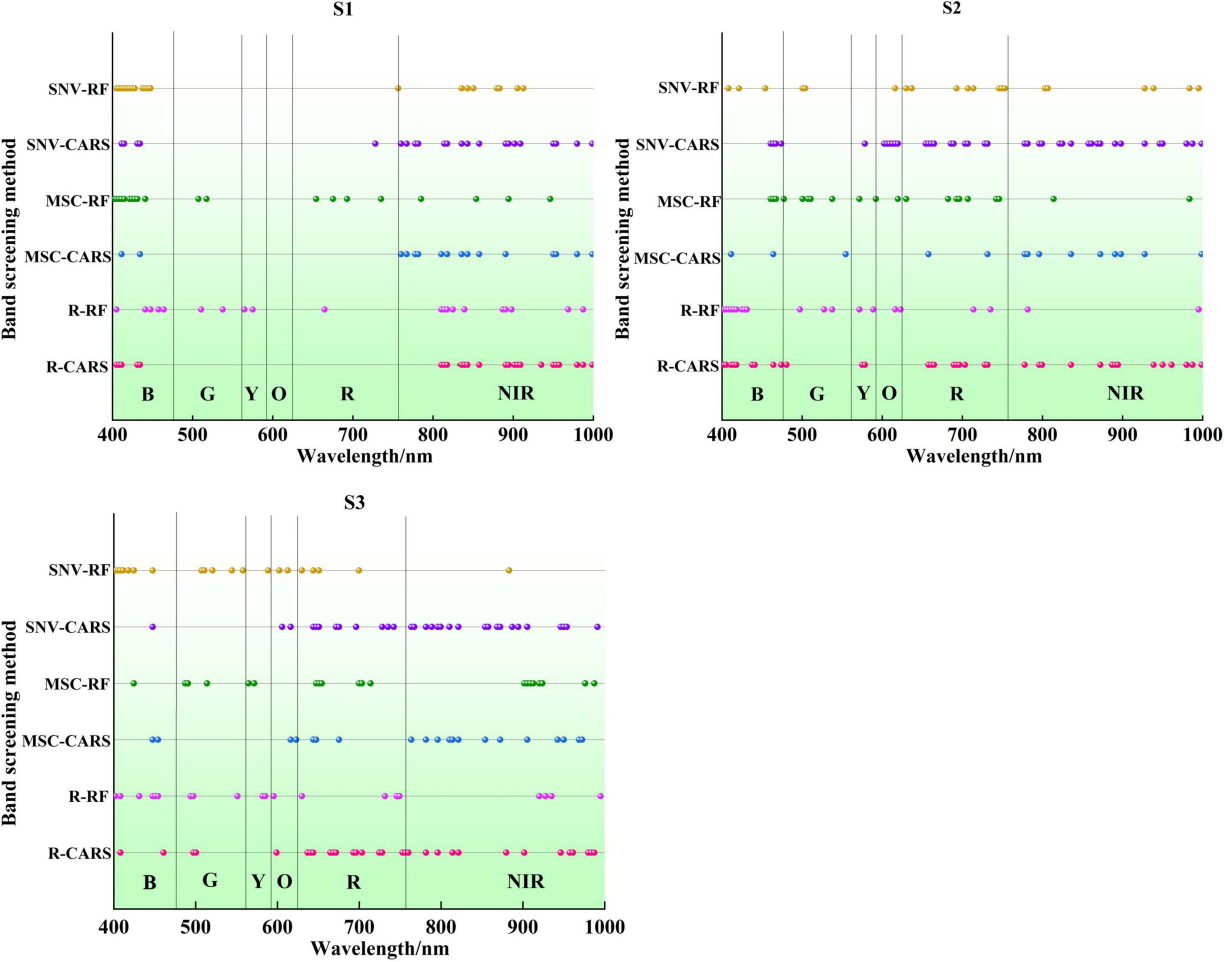
Distribution of feature band screened by different feature band screening algorithms.

The results of band selection processed by CARS and RF are shown in **Figure 6**, which shows the influence of different selection methods and potato growth stages on the results. Utilizing RF for band selection involves choosing the top 20 bands based on their selection probability, representing 11.36% of all bands. For S1, the feature band identified by the RF band selection methods across in the three types of spectral data was mainly concentrated in the near infrared region of 760-1000 nm, with a smaller distribution in the visible light region of 400-475 nm. For S2, the feature band screened by the RF band selection methods on the three types of spectral data are distributed in both visible light and near-infrared regions, with a more scattered distribution. Fewer feature band selected by RF on the three types of spectra are distributed in the near-infrared region. For S3, the distribution of the feature band selected by RF on the three types of spectra follows the same pattern as in the S2 stage, with a few distributed in the near-infrared region.

For S1, the feature band selected by CARS based on R, MCS, and SNV spectral data are 25, 16, and 22, accounting for 14.20%, 9.09%, and 12.50% of the total bands, respectively. For S2, the feature band screened by the CARS band selection methods on the three types of spectral data are distributed in both visible light and near-infrared regions, with a more scattered distribution. CARS selects 35, 14, and 40 the feature band based on R, MCS, and SNV spectral data, accounting for 19.89%, 7.95%, and 22.73% of the total bands, respectively. For S3, the distribution of the feature band selected by CARS on the three spectra is quite similar, mainly concentrated between 550-830 nm. The feature band selected based on R, MCS, and SNV spectral data are 31, 20, and 31, accounting for 17.61%, 11.36%, and 17.61% of the total bands, respectively. Both band selection methods reduce the spectral dimensions across the three spectral data types, simplifying the model and reducing the computational load.

### 3.4 Potato LWC estimation models

**Figure 7:**
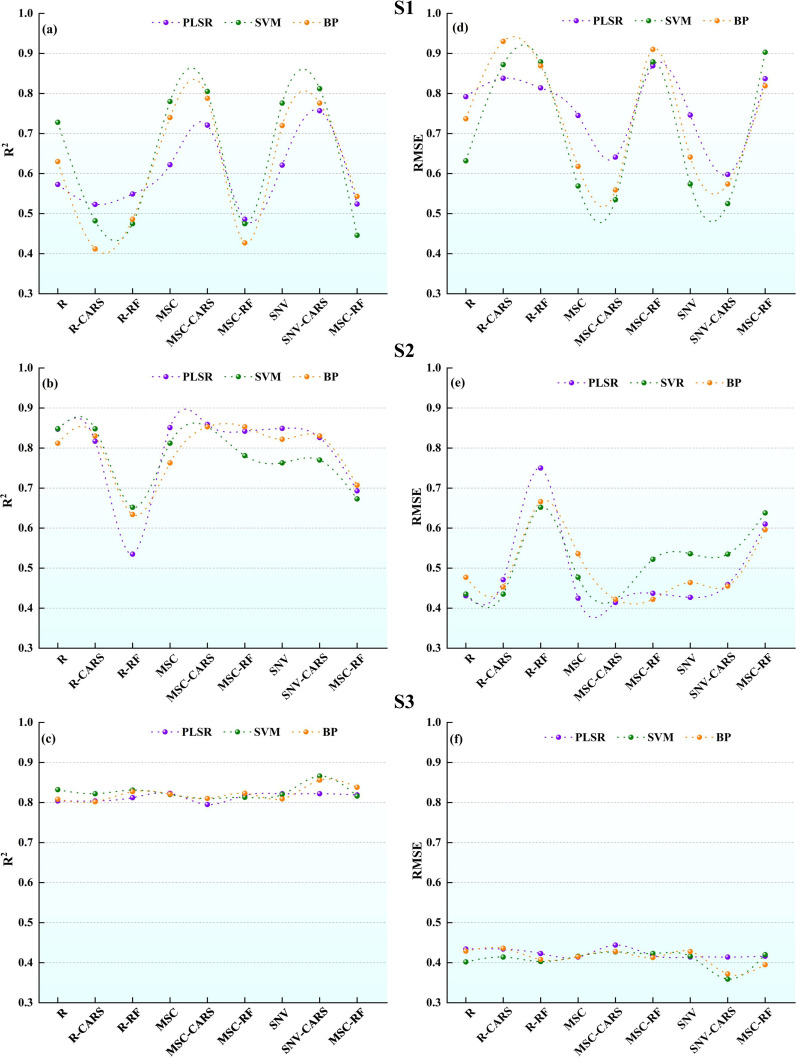
Comparison of potato LWC estimation accuracy using three machine learning models: **(A-C)** R^2^, **(D-F)** root mean square error (RMSE).

We used machine learning methods, such as PLSR, SVR, and BP, to develop estimation models for LWC. To achieve more accurate predictions, three different growth stages were modeled independently. In addition, we analyzed the impacts of the full-band and the feature band as variables in the models. 75% of the data is randomly selected to construct the modeling set, and the rest was used as the test set. **Figure 7** shows the results of various models on the modeling set.

For S1, the PLSR models show the accuracies ranging from 0.49 to 0.76 for R^2^ and 0.60 to 0.87 for RMSE. The MSC-CARS-PLSR model demonstrates the highest R^2^ and the smallest RMSE, while the SNV-RF-PLSR model has the lowest R^2^ and the largest RMSE. Similarly, the SVR models have accuracies ranging from 0.45 to 0.81 for R^2^ and 0.53 to 0.90 for RMSE. The accuracies of the BP models ranged from 0.41 to 0.79 for R^2^ and from 0.56 to 0.93 for RMSE. Within this context, the SNV-CARS-BP model demonstrates the highest R^2^ and the smallest RMSE, whereas the R-CARS-BP model exhibits the lowest R^2^ and the largest RMSE. In the models constructed using raw spectral data R, the full-band models exhibit better modeling performance than the models constructed from selected the feature band. For the models established with spectral data transformed by SNV and MSC, those developed with the feature band selected by CARS outperform the full-band models. However, the performance of the models based on the feature and selected by RF is inferior to that of the full-band models. In addition, after applying SNV and MSC transformations to the original spectral data R, the modeling effect was improved to varying degrees. In particular, the MSC-CARS-SVR model achieves the best modeling effect at the S1 stage.

For S2, the modeling accuracy of the PLSR models ranges from 0.54 to 0.86 for R^2^ and from 0.41 to 0.75 for RMSE. Among the models, the SNV-CARS-PLSR model has the highest R^2^ and the smallest RMSE, while the R-RF-PLSR model has the lowest R^2^ and the highest RMSE. The modeling accuracy of the SVR models ranges from 0.65 to 0.85 for R^2^ and 0.42 to 0.65 for RMSE. In this case, the SNV-CARS-SVR model presents the highest R^2^ and the smallest RMSE, while the R-RF-SVR model indicates the lowest R^2^ and the highest RMSE. Additionally, the modeling accuracy of the BP models ranges from 0.63 to 0.85 for R^2^ and 0.42 to 0.67 for RMSE, with the SNV-CARS-BP model achieving the highest R^2^ and the smallest RMSE and the R-CARS-BP model recording the lowest R^2^ and the highest RMSE. In models established based on the spectral data after MSC transformation, those using the full-band for modeling showed better performance than those using models built with selected the feature band. In the models established based on the original spectral data R and spectra after SNV transformation, models constructed using bands selected by CARS had better performance than those built using the full-band. However, models built with bands selected by RF performed worse than models established with the full-band. Notably, three models established based on data processed by SNV-CARS all achieved optimal predictive performance, with the SNV-CARS-PLSR model emerging as the most accurate at the S2 stage.

For S3, the PLSR model’s R^2^ modeling accuracy falls between 0.80 and 0.82, with an RMSE ranging from 0.41 to 0.44. Importantly, the MSC-CARS-PLSR model demonstrates the highest R^2^ and the smallest RMSE, while the SNV-CARS-PLSR model exhibits the lowest R^2^ and the largest RMSE. Likewise, the SVR model’s R^2^ modeling accuracy spans from 0.81 to 0.87, with an RMSE between 0.36 and 0.43. Once more, the MSC-CARS-SVR model yields the highest R^2^ and the smallest RMSE, with the SNV-CARS-SVR model recording the lowest R^2^ and the highest RMSE. In the case of the BP models, the R^2^ modeling accuracy ranges from 0.80 to 0.86, with an RMSE between 0.37 and 0.44. In this context, the MSC-CARS-BP model demonstrates the highest R^2^ and the smallest RMSE, while the R-CARS-BP model exhibits the lowest R^2^ and the largest RMSE. Comprehensive analysis reveals that all the models developed during the S3 stage show an R^2^ above 0.80 in the prediction set, with minor variations in R^2^ and RMSE among the models. Remarkably, three models established based on data processed by MSC-CARS all achieved optimal predictive performance, with the MSC-CARS-SVR model boasting the highest prediction accuracy during the S3 stage.

**Figure 8:**
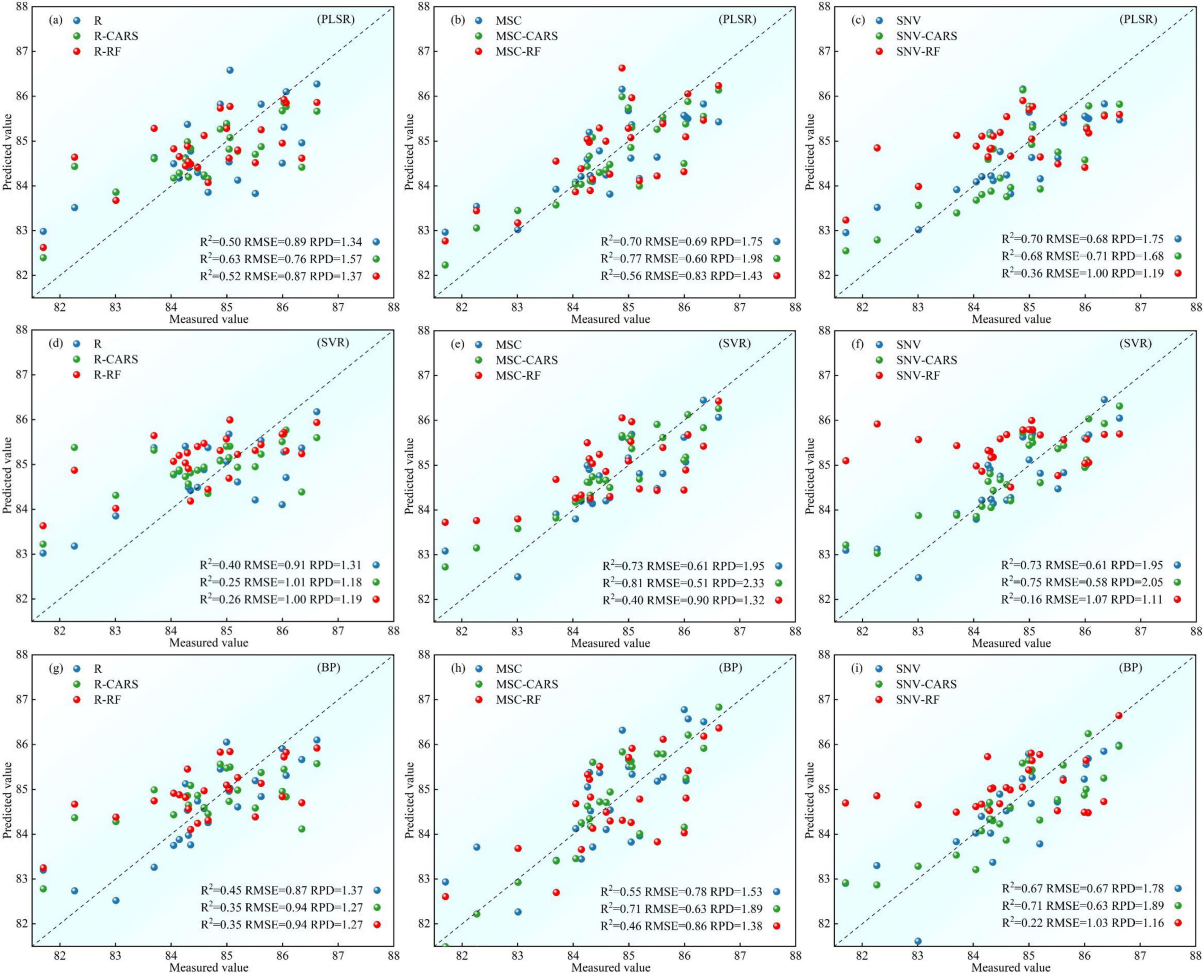
Comparison of test set accuracy of potato S1 phase LWC estimation using three machine learning models: **(A-C)** PLSR models, **(D-F)** SVR models, **(G-I)** BP models.

The potato LWC estimation models should demonstrate high fit universality and repeatability. Accordingly, we further evaluated the accuracy of each growth stage models on the test set. For S1, **Figure 8** presents the validation results on the test set for each model. Among them, the nine models based on the R spectrum data show poor test fitting on the test set, with R^2^ ranging from 0.25 to 0.63, RMSE ranging from 0.76 to 1.01, and RPD ranging from 1.18 to 1.57. It is noteworthy that, when models being built with the feature band, only the PLSR models surpasses the full-band models. Conversely, the test performances of the SVR and BP models are inferior to the full-band models. In contrast, the nine models based on the MSC spectral data demonstrate good test fitting on the test set, with R^2^ ranging from 0.40 to 0.81, RMSE ranging from 0.51 to 0.90, and RPD ranging from 1.32 to 2.33. Models with the feature band selected by CARS demonstrate superior test performance compared to those of the full-band, while models with the feature band selected by RF show inferior test performance. Concerning the nine models based on the SNV spectral data, the test set R^2^ ranges from 0.16 to 0.75, RMSE ranges from 0.58 to 1.07, and RPD ranging from 1.11 to 2.05. Models established using the feature band display a test performance similar to that of the MSC spectral data, where the CARS models demonstrate better test performance than those of the full-band, and the RF models demonstrate inferior test performance. Comparing the test effects of various models for the potato S1 phase, the MSC-CARS-SVR model demonstrates the best test set performance, with R^2^ = 0.81, RMSE = 0.51, and RPD=2.33, indicating its potential for potato S1 phase LWC content inversion.

**Figure 9:**
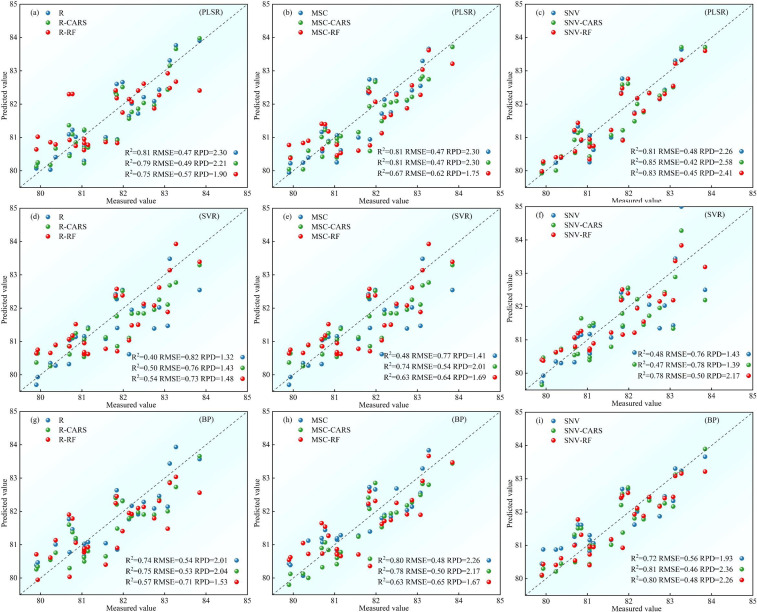
Comparison of test set accuracy of potato S2 phase LWC estimation using three machine learning models: **(A-C)** PLSR models, **(D-F)** SVR models, **(G-I)** BP models.

For S2, **Figure 9** presents the validation results of the test set samples on each model. In the group of nine models based on the R spectrum data, the test set R^2^ ranges from 0.40 to 0.81, with RMSE ranging from 0.47 to 0.82, and RPD ranging from 1.32 to 2.30. Remarkably, the PLSR models with the full-band surpass the those with the feature band in terms of test performance. For the SVR and BP models, the test performance of the models using the feature band selected by the CARS algorithm is superior to the full-band models. Conversely, the RF models demonstrates inferior test performance. Concerning the nine models relying on the MSC spectral data, the test set R^2^ ranges from 0.48 to 0.81, RMSE ranges from 0.47 to 0.77, and RPD ranging from 1.41 to 2.30. Solely the feature band PLSR models outperforms the full-band models in terms of test performance, while the SVR and BP lag behind. Additionally, the nine models based on the SNV spectral data display satisfactory test fitting on the test set, with R^2^ ranging from 0.47 to 0.85, RMSE ranging from 0.42 to 0.78, and RPD ranging from 1.39 to 2.58. Specifically, only the SVR models constructed using the feature band selected by CARS exhibits lesser test performance than the full-band models. Conversely, the remaining models using the feature band demonstrate superior test performance. By comparing the test effects of various models for the potato S2 phase, the SNV-CARS-PLSR model emerges with the most optimal test set performance, boasting R^2^ = 0.85, RMSE = 0.42, and RPD = 2.58, signifying its effectiveness in inverting the potato S2 phase LWC content.

**Figure 10:**
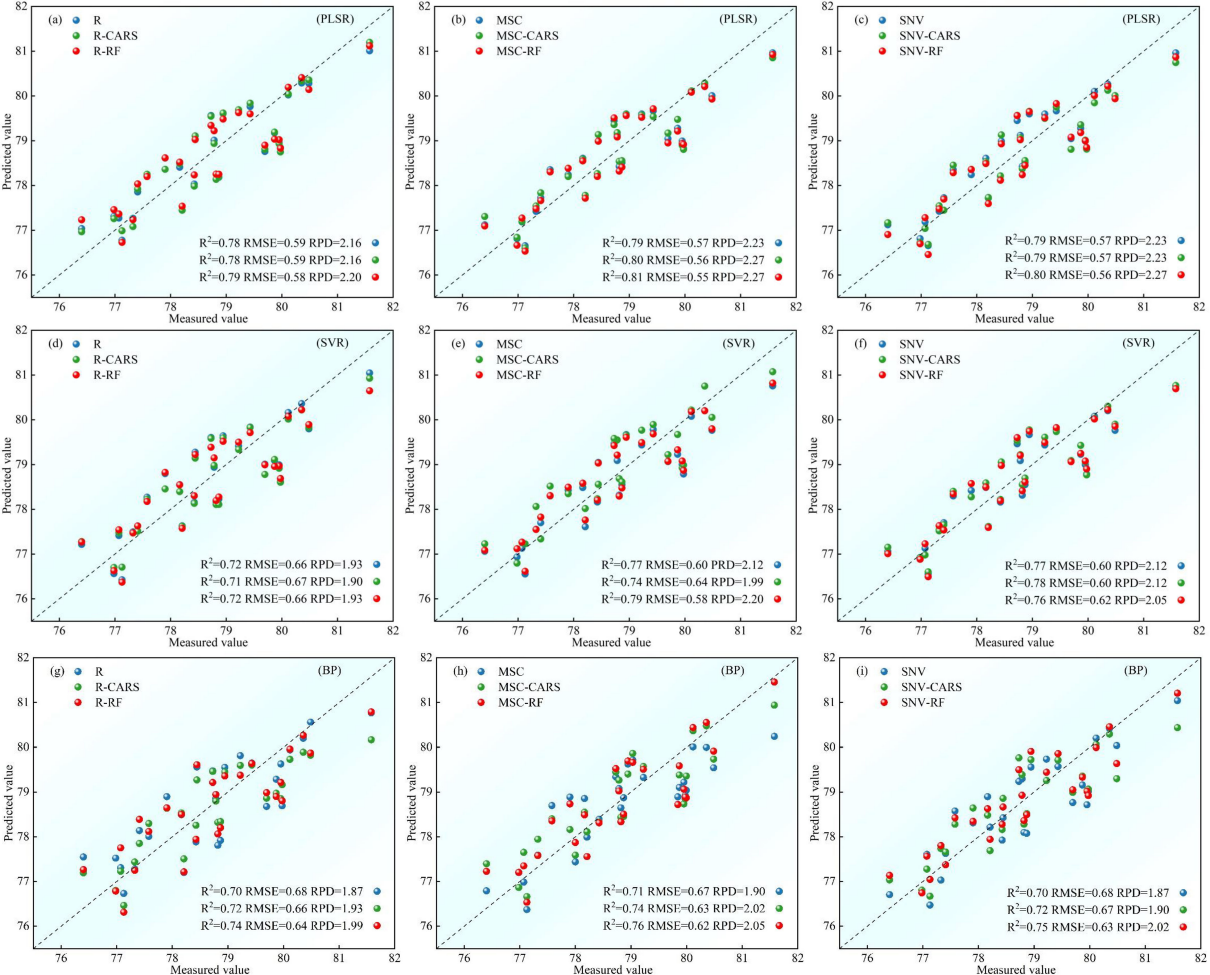
Comparison of test set accuracy of potato S3 phase LWC estimation using three machine learning models: **(A-C)** PLSR models, **(D-F)** SVR models, **(G-I)** BP models.

For S3, **Figure 10** presents the validation results of the test set samples on each model. All models exhibit a test set R^2^ above 0.70, RMSE below 0.68, and RPD above 1.87. Particularly, among models constructed with the feature band, the test performance of the SVR models, utilizing the feature band selected by CARS, is inferior to the full-band models for the R and MSC spectral data. However, in all other cases, the test performance of the models constructed using the feature band surpasses that of the full-band models. When comparing the test effects of various models for the potato S3 phase, the MSC-RF-PLSR model stands out with the most superior test set performance, boasting an R^2^ of 0.81, RMSE of 0.55, and RPD of 2.58. This model effectively monitors the LWC content of potato S3 phase.

Overall, it can be seen that there is no “omnipotent” model to make the most accurate prediction of LWC in different growth stages, so modeling the different growth stages separately is a better choice. For S1, S2 and S3 stages, the most appropriate prediction models are MSC-CARS-SVR, SNV-CARS-PLSR and MSC-RF-PLSR respectively.

### 3.5 Spatial distribution of field potato LWC

**Figure 11:**
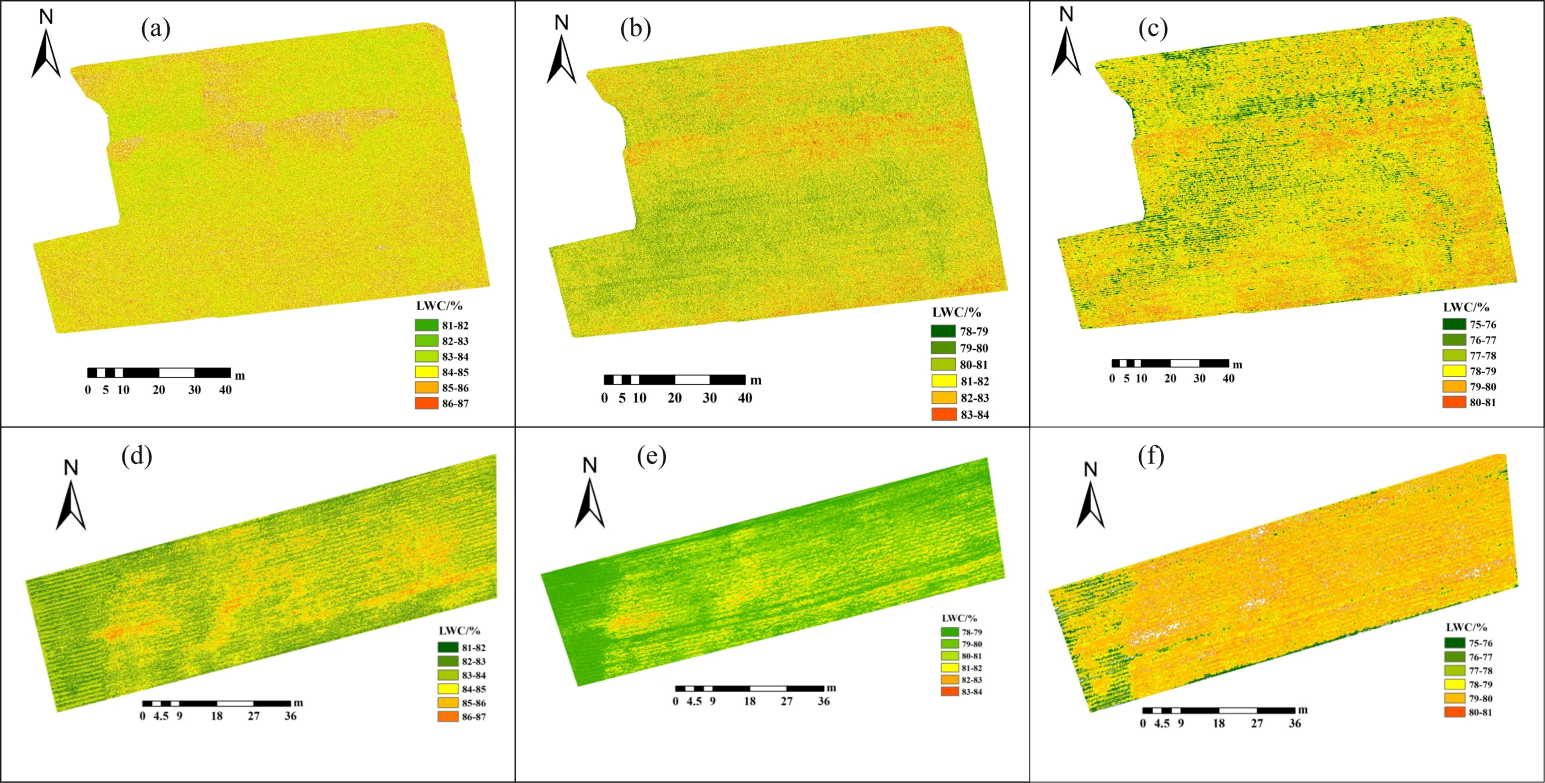
Spatial distribution of potato LWC based on the optimal estimation models: **(A-C)** S1-S3 period in 2021, **(D-F)** S1-S3 period in 2022.

Utilizing the best LWC estimation models outlined in Section 3.4, **Figure 11** displays the spatial distribution map of LWC for S1, S2, and S3 in 2021 and 2022. Potato LWC levels exhibit notable variations across diverse plots and growth stages, with the S1 stage displaying higher LWC content than the S2 and S3 stages. Additionally, noticeable disparities in potato LWC are observed among different regions within the same plot. Potato plants demonstrate a decrease in LWC during the S3 phase when contrasted with S2. During the S3, the plants prioritize nutrient transfer to the tubers to facilitate tuber growth and starch accumulation. Therefore, foliage may undergo a reduction in water content during this phase to fulfill the nutritional requirements for tuber development. It can be observed that the distribution of LWC in the map is uneven. Some regions, shown in light yellow or orange, indicate higher LWC and may be in vegetation growth and sufficient moisture content. In contrast, dark green regions mean lower LWC and may be under-irrigated. The uneven distribution can indicate which areas should be prioritized for irrigation. By utilizing this map, farmers and managers can pinpoint regions in need of precision irrigation, potentially increasing crop yield achieving the goal of water-saving irrigation.

## 4 Discussion

### 4.1 Spectral transformation and feature selection for potato LWC

Mathematical transformation to the original spectrum is essential to improve data quality and modeling accuracy. In our study, we applied MSC and SNV transformations to the potato canopy hyperspectral data, which resulted in a significant improvement in the correlation between spectral data and potato LWC. This result is consistent with the research results of Sun et al. (2017) and Chen et al. (2020b), who reported improved correlation values after mathematical transformations of the spectra of purple sweet potatoes and apple leaves. Hyperspectral data contain a significant number of bands, many are irrelevant to potato LWC and make little contribution to the accuracy of the estimation model. Therefore, the full-band modeling is not the best choice. The selection of the feature band handled by CARS and RF helps to reduce data redundancy and improve modeling performance, the result consistent with that of Sudu et al. (2022) and Liu et al. (2020).

### 4.2 Hyperparameter selection for machine learning models

**Figure 12:**
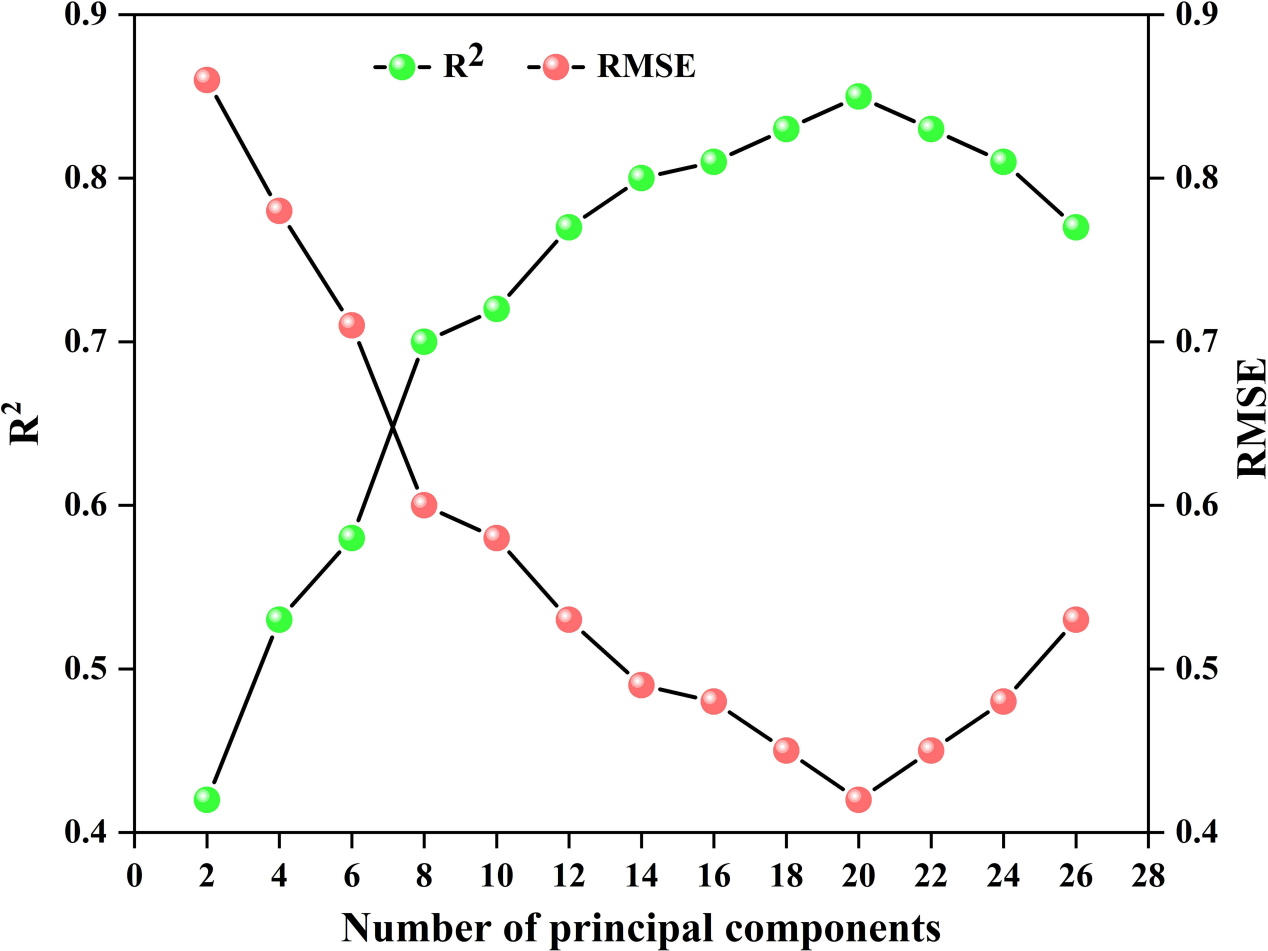
Variation of R^2^ and RMSE during the optimization of the number of principal components in the SNV-CARS-PLSR model at the potato S2 stage, with 40 input features.

The performance of machine learning models is heavily influenced by the selection of hyperparameters, and optimizing these hyperparameters directly impacts both the generalization capability and predictive accuracy of the models. To ensure optimal model performance, this study employs grid search and cross-validation to optimize the hyperparameters of PLSR, SVR, and BP. For the PLSR model, the number of principal components is a crucial hyperparameter that influences the model’s complexity and generalization ability. Taking the optimization of the number of principal components in the SNV-CARS-PLSR model during the potato S2 stage as an example, **Figure 12** demonstrates a clear relationship between the number of principal components and the model’s R^2^ and RMSE values. As the number of principal components increases from 2 to 20, the R^2^ value rises while the RMSE decreases, reaching a peak R^2^ of 0.85 at 20 components, with the RMSE minimizing at 0.48. However, beyond 20 components, the R^2^ value begins to decline, and the RMSE increases, leading to a decrease in model performance. Therefore, the optimal number of principal components for this model is 20.

**Figure 13:**
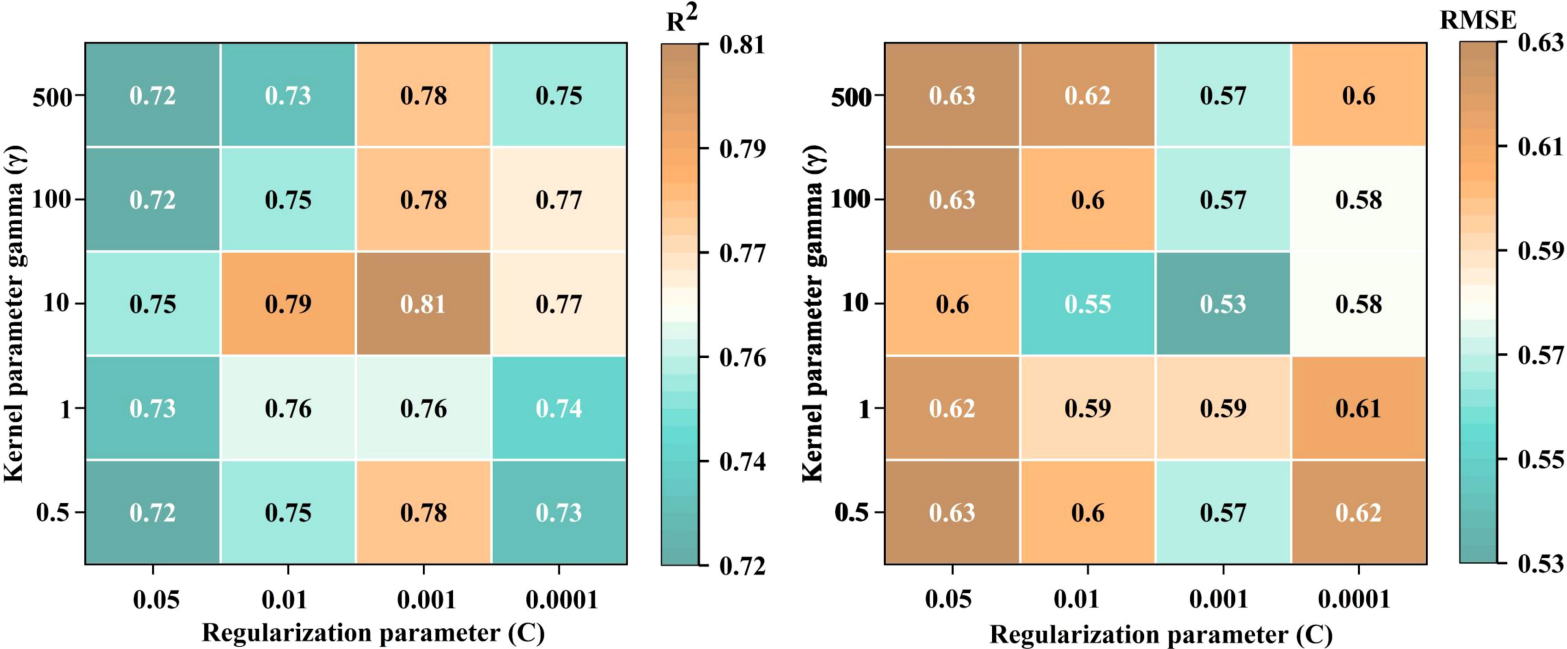
Variation of R^2^ and RMSE during the optimization of hyperparameters text C and γ in the MSC-CARS-SVR model at the potato S1 stage.

For the SVR model, the MSC-CARS-SVR model at the potato S1 stage was used as an example to optimize the hyperparameters C and γ. As shown in **Figure 13**, the RMSE exhibits a clear trend with varying values of C and γ. When C = 10 and γ = 0.001, the model achieves the lowest RMSE (0.53), indicating optimal generalization ability. Additionally, **Figure 13** illustrates the variation in R^2^ values corresponding to different C and γ parameters. Consistent with the RMSE heatmap, the model reaches its highest R^2^ value of 0.81 at C = 10 and γ = 0.001, further confirming the best model fit under this parameter combination. Therefore, the optimal parameters for this model are C = 10 and γ = 0.001.

For the BP model, the MSC-CARS-BP model during the potato S3 stage was used as an example, and grid search was employed to identify the optimal constant *α*. As shown in **Table 2**, variations in the number of *q* and the constant *α* resulted in significant fluctuations in the model’s R^2^ and RMSE values. Notably, when *q* = 7 and *α* = 3, the model achieved the highest R^2^ value of 0.86 and the lowest RMSE of 0.37, indicating optimal model fit. However, an excessive number of nodes may cause a decrease in R^2^ and an increase in RMSE, potentially due to network complexity leading to overfitting. Therefore, the optimal parameters for this model are *q* = 10 and *α* = 3.

**Table 2:** Variation of *R* ^2^ and RMSE during the optimization of BP model hyperparameters *C* and γ.

q	α	$R^{2}$	RMSE
5	1	0.83	0.41
6	2	0.85	0.39
7	3	0.86	0.37
8	4	0.84	0.40
9	5	0.83	0.41
10	6	0.83	0.41
11	7	0.80	0.44
12	8	0.79	0.45
13	9	0.77	0.47
14	10	0.77	0.47

*q* represents the number of nodes in the hidden layer of the BP neural network, and *α* is a constant within the range [1, 10]. Using the MSC-CARS-BP model at the potato S3 stage as an example, the number of input features is 20.

By optimizing the hyperparameters of the PLSR, SVR, and BP neural network models, this study successfully enhanced the prediction accuracy and generalization ability of each model. The results demonstrate that proper hyperparameter tuning is crucial for improving model complexity and predictive performance. Future research could explore more complex model structures and optimization algorithms to further enhance model performance.

### 4.3 Impact of different machine learning algorithms on potato LWC estimation performance

The spectral features of crops are intricately linked to their growth stages, health status, and external environment. These spectral curves undergo distinctive changes across different growth stages (Panigrahi and Das, 2018). A single model can’t make accurate predictions for the LWC of the three growth periods. In order to solve this problem, we established LWC estimation models for tuber formation, growth, and starch accumulation stage, respectively - three pivotal growth stages of potatoes. Upon analyzing the potato LWC estimation results for these stages, variations in model accuracy are evident. The model accuracy exhibits a trend of initial increase and subsequent decrease as the growth stages progress, possibly attributed to changes in canopy structure, biomass accumulation, and the distribution of leaf water content as potato growth stages advance, leading to varied predictive performance. Notably, PLSR models emerge as good choice for estimating LWC during the growth and starch accumulation stage. In contrast, SVR models proves better during the tuber formation stage. Primarily, this preference for PLSR is justified by the challenge of multicollinearity among the hyperspectral bands, wherein PLSR demonstrates superior capability in handling multicollinearity compared to SVR and BP algorithms. Additionally, the small sample size of this study suggests that SVR and BP may be susceptible to overfitting. PLSR generally requires less data for small sample sizes, making models established by PLSR more robust than those derived from SVR and BP.

### 4.4 Application of potato LWC distribution maps in adjusting precision irrigation strategies

During potato cultivation, LWC is a key indicator of crop water status and plays a critical role in growth (Suyala et al., 2024; Zununjan et al., 2024). Analysis of LWC data derived from UAV-based hyperspectral inversion shows that the water requirements of potatoes vary significantly at different developmental stages. This finding highlights the importance of developing precise irrigation strategies. The spatial distribution map of LWC can guide irrigation in three main aspects. First, irrigation regulation can be based on LWC variability. As shown in **Figure 11**, certain regions have low LWC (e.g., yellow and orange areas), indicating insufficient soil moisture, which may limit crop growth. In these regions, timely increases in irrigation are needed to maintain adequate moisture, promoting root growth and normal tuber development. In contrast, regions with higher LWC (e.g., green areas) should receive reduced irrigation to avoid wasting water and potential soil salinization. Second, precision irrigation can be implemented through zoning management. By using spatial analysis of hyperspectral images, the field can be divided into multiple zones. Each zone can then receive precision drip irrigation based on its LWC levels. The drip irrigation system allows flexible adjustment of water supply according to actual soil moisture needs. This zoning management ensures that each area receives an appropriate water supply during the reproductive period, realizing the principle of ‘water supply according to demand.’ Finally, irrigation timing should be dynamically adjusted. Time-series analysis of hyperspectral images reveals that potato water demand fluctuates at different growth stages. During S1 and S2 stages, water demand is higher, especially in areas with lower LWC (see **Figure 11**). In these cases, irrigation frequency and volume should be increased to prevent water shortage. However, during the S3 stage, while water remains important, irrigation should be moderated to prevent excess moisture from inhibiting starch accumulation. Future research will integrate hyperspectral data with soil moisture sensors and meteorological data to further optimize water usage. This will enhance automated irrigation management, contributing to improved potato productivity and quality.

The study aims to develop potato LWC estimation models based on the feature band. Despite achieving impressive accuracy, it faces challenges during training with the machine learning algorithm. This is primarily due to the limited number of test samples, resulting in overfitting and degradation of the model. To overcome this limitation, it is essential to expand the scope of the investigation by increasing the number of sample, period, and potato varieties to validate and enhance the model’s applicability. Future research will focus on acquiring more data in more sites, leveraging a larger sample size to enhance the model’s robustness and accuracy.

## 5 Conclusion

The rapid measurement of LWC in the canopy allow farmers know the water distribution of potatoes in the field, so as to formulate water-saving irrigation strategies. In this study, UAV was used as a platform to efficiently collect hyperspectral data of potato canopy at the field scale, and LWCs were actually measured on the ground. After mathematical transformation and the feature band selection, the relationships between LWC and hyperspectral data were analyzed, and the estimation models of LWC were modeled by machine learning. In order to make the model prediction results more accurate, we modeled the LWC of the three main growth stages of potato. The original spectral data underwent two mathematical transformations: MSC and SNV. The methods of the feature band selection contained CARS and RF algorithms. Our modeling approaches included PLSR, SVR, and BP. The investigation yielded the following pivotal findings:

1. Applying MSC and SNV mathematical transformations to the potato canopy hyperspectral data significantly enhanced the correlation between the spectral data and potato LWC. Specifically, the correlation coefficient based on R increased by -0.50 to 0.35 under MSC and by -0.30 to 0.40 under SNV.
2. The band extraction algorithms CARS and RF effectively selected the most relevant bands and reduced data redundancy. Feature band selected by RF represented approximately 11.36% of all bands, while those selected by CARS accounted for 7.95% to 22.73%.
3. The accuracy of the models varied at different stages of potato growth. The optimal models for estimating LWC in stages S1 to S3 were MSC-CARS-SVR, SNV-CARS-PLSR, and MSC-RF-PLSR. These three models consistently provided stable and accurate estimation of potato LWC.

## Data Availability

The raw data supporting the conclusions of this article will be made available by the authors, without undue reservation.
